# Genetic variation in the ovine *KAP22-1* gene and its effect on wool traits in Egyptian sheep

**DOI:** 10.5194/aab-65-293-2022

**Published:** 2022-08-05

**Authors:** Ahmed M. Sallam, Aymen A. Gad-Allah, Essam M. Albetar

**Affiliations:** 1 Animal and Poultry Division, Desert Research Center, 1st Mathaf El-Mataryia, 11735, Cairo, Egypt; 2 Department of Wool Technology and Production, Desert Research Center, 1st Mathaf El-Mataryia, 11735, Cairo, Egypt

## Abstract

The objective of this study was to investigate the genetic polymorphisms in the keratin-associated protein (*KAP22-1*) gene in Barki (n=206), Rahmani (n=28) and Ossimi (n=28) as the three major sheep breeds in Egypt. Subsequently, the detected variants were correlated with important wool traits. The traits included greasy fleece weight (GFW, g), staple length (SL, cm), prickle factor (PF, %), medullated fiber (MF, %), fiber diameter (FD, µm), crimp percentage (CR, %) and the standard deviation of FD (SD${}_{\mathrm{fd}}$, µm), as well as the subjectively assessed traits of kemp score (KS), handle grade (HG), greasy color grade (GCG), bulk grade (BG), luster grade (LG) and staple structure (SST). Animals were genotyped by polymerase chain reaction (PCR) – single strand conformation polymorphism (SSCP). Five SSCP banding patterns representing three different nucleotide variants (A, B and C) were detected. DNA sequencing confirmed three single nucleotide polymorphisms (SNPs). Animal age significantly affected GFW (P=0.007), SD${}_{\mathrm{fd}}$ (P=0.006), SL (P=0.002), CR (P=0.006), KS (P=0.001), LG (P=0.006) and SST (P=0.013). Likewise, the breed had a significant effect on all studied traits except HG and BG, which was not significant. Results showed significant associations between the *KAP22-1* variants and CR (P=0.01), SL (P=0.012), KS (P<0.001) and GCG (P=0.01). Interestingly, animals with BB genotypes tended to produce more wool yield (1163.63±65.91 g) with high SL (8.38±0.20 cm), CR (8.38±0.21 %) and KS (1.98±1.88). Results of this study strongly recommend the *KAP22-1* gene as a candidate gene for wool production traits in Egyptian sheep, with new useful insights into the visually assessed wool traits. The identified genetic markers may be incorporated into breeding strategies and genetic improvement programs of wool traits in Egyptian sheep.

## 1 Introduction

Globally, sheep contribute significantly to the animal production industry with various products including, milk, meat and wool. The total sheep population in Egypt is about 5.1 million heads that produce about 11 217 metric tons of greasy fleece annually (FAOSTAT, https://www.fao.org/faostat/en/, last access: 22 November 2021). In Egypt, Barki, Rahmani and Ossimi are considered the three major sheep breeds (Galal et al., 2005), representing about 65 % of the Egyptian sheep population (Elshazly and Youngs, 2019). They are distributed along the western Mediterranean coastal region, the middle of Egypt and the northern Nile delta, playing an important role in the livelihood of large portions of Egyptians (Sallam, 2019). Egyptian sheep are considered a source of low-quality woolens with coarse fleece and include some kemp (Helal et al., 2019), which may be appropriate only for handmade carpets. Under extensive conditions where high lamb production is not allowed, wool could be an important source of increasing the impact of raising these animals (Thomas, 2015). Several attempts were made to improve wool production of local breeds by crossing with imported breeds, such as Merino, Romanov and Finn sheep (Almahdy et al., 2000).

Obviously, wool is the only source of woolen products and contributes significantly to the worldwide textile industry. Generally, producing high-quality wool will maximize the profitability of the wool industry. Wool characteristics greatly affect wool's suitability for processing, such as fiber diameter, staple length and strength, color, and other visually assessed traits (Mortimer et al., 2009; Helal et al., 2019). Wool traits are quantitative traits that are affected by both environment and the animal genotype. Additionally, multiple genes contribute to traits with different biological mechanisms. Understanding the genetic contribution and biological functions of a particular trait is essential for subsequent selection and genetic improvement programs (Oldenbroek and Van der Waajj, 2015; Noelle and Chunhua, 2015).

Recently, the advent of genotyping technologies enabled breeders to pinpoint the genetic architecture of economically important traits (Sallam et al., 2018). Over the last 2 decades, multiple candidate genes harboring single-nucleotide polymorphisms (SNPs) for various production traits were reported in livestock (Wilkening et al., 2009). Association analysis using SNPs is the most effective approach to identify genetic markers related to a trait of interest (Zhu and Zhao, 2007). SNPs screen the candidate genes, which may be biologically related to the desired trait, for putative mutations (Patnala et al., 2013). Subsequently, these mutations are genotyped in a group of individuals and correlated with certain phenotypes (Kwon and Goate, 2000). Therefore, SNPs became the most common genetic marker used in selection and evaluation programs in livestock (Dominik et al., 2021). Conversely, scarce information is available about the genetic background of wool traits as few genes (e.g., *KAP1.1*, *KAP1.3* and *KAP6*) were identified for wool traits in Egyptian sheep (Farag et al., 2018; Sallam et al., 2021).

*KAP22-1* is a part of the keratin-associated proteins (KAPs), which are the main structural proteins of wool and hair fibers (Parsons et al., 1994; Powell and Rogers, 1997). The physicomechanical properties of the wool fibers are determined by these proteins (Powell and Rogers, 1997). The KAPs contain high levels of cysteine, glycine and tyrosine, which are predominantly located in the wool fiber cortex. It was reported that different numbers of KAPs are found in different wool types, which raises questions about their function in identifying wool fiber characteristics (Ullah et al., 2020b). The gene is located at chromosome 1, spanning 391 base pairs and consisting of three exons (Kinsella et al., 2011). The gene was identified as a candidate gene for wool traits (Li et al., 2017), harboring putative SNP markers for wool traits (Gong et al., 2016). The objective of this study was to investigate the genetic polymorphisms in the *KAP22-1* gene and then correlate these variants with important wool traits in three major sheep breeds in Egypt.

## 2 Materials and methods

### 2.1 Animals and phenotypes

All animal procedures included in the current study were approved by the animal breeding committee at the Desert Research Center (DRC). A total of 262 individuals from three different Egyptian sheep breeds were included in the study. Blood and wool samples for Barki sheep (n=206) were collected from the Maryout Research Station, DRC, located in the Egyptian north coastal zone, while Rahmani (n=28) and Ossimi (n=28) samples were obtained from commercial farms located in the northern Nile delta of Egypt. For each animal, wool samples were collected from the mid-side region to be used in the subsequent assessments. Greasy fleece weight (GFW, kg) was recorded at yearling shearing. Staple length (SL, cm), prickle factor (PF%; the percentage of fibers of diameter >30 µm), medullated fiber (MF%), fiber diameter (FD, µm), crimp percentage (CR%) and the standard deviation of FD (SD${}_{\mathrm{fd}}$) were recorded for each individual independently. The subjective traits included kemp score (KS), handle grade (HG), greasy color grade (GCG), bulk grade (BG), luster grade (LG) and staple structure (SST). These traits were measured following the grading method of Dry (1935), El-Gabbas (1994) and Helal et al. (2019). For each fleece, one experienced grader has taken a composite representative sample. HG, GCG, BG and LG had five grades, while KS had four grades. All Rahmani wool samples with brown or black pigments were excluded when assessing GCG as it basically assesses the degree of greasy whiteness. Grade 1 was assigned to wool samples that have no kemp (KS), are yellow (GCG) and are the harshest (HG) wool with the least luster (LG) and compressibility (SST), whereas grade 4 (i.e., KS only) or 5 (i.e., the other traits) has dense kemp fibers, is perfect white, and is the softest and extremely lustrous with compressibility (El-Gabbas and El-Wakil, 2016). Phenotypic characteristics of wool production traits collected from animals included in the study are presented in Table 1.

**Table 1:** Phenotypic characteristics of wool production traits in Egyptian sheep.

Traits${}^{a}$	Breeds${}^{b}$											
	Barki (206)				Rahmani (28)				Ossimi (28)			
	Av.	SD	Min.	Max.	Av.	SD	Min.	Max.	Av.	SD	Min.	Max.
GFW, g	1128.22	379.38	300	1900	NA	NA	NA	NA	NA	NA	NA	NA
SL, cm	8.01	1.75	4.33	13.00	8.61	1.41	6.5	11	7.15	2.06	4	12.66
FD, µm	26.55	4.48	18.35	49.68	25.83	3.11	18.85	29.88	30.28	3.43	23.28	38.76
SD${}_{\mathrm{fd}}$, µm	15.26	8.13	7.37	48.64	11.56	3.14	6.95	9.13	14.66	4.55	8.06	23.86
MF%	13.69	9.76	0.6	42.4	13.24	5.61	5.2	25.4	10.86	5.57	4.6	25.6
PF%	26.00	3.88	10.11	63.18	21.1	10.46	1.4	40.6	33.34	10.69	49.2	12
CR%	1.11	0.38	0.25	1.65	0.78	0.14	0.48	1.01	0.62	0.15	0.9	0.39

${}^{a}$ GFW: greasy fleece weight (g); SL: staple length (cm); FD: fiber diameter (µm); SD${}_{\mathrm{fd}}$: the standard deviation of FD; MF: modulated fiber; PF: pickle factor; CR: crimp percentage %. ${}^{b}$ Av.: average; SD: standard deviation; Min.: minimum; Max.: maximum; NA: not available.

### 2.2 DNA extraction and genotyping

Blood samples were collected from the jugular vein of each animal in the study. Genomic DNA was extracted from the whole blood samples using Intronbio (commercial kits, Germany) following the manufacturer protocol. Finally, DNA samples were stored in a laboratory freezer at -20 ${}^{\circ }$C. The specific forward and reverse primers 5${}^{\prime }$-TATGAGTGCAACAGTGACTG-3${}^{\prime }$ and 5${}^{\prime }$-CCATGTTTTGAATAGACAAGC-3${}^{\prime }$ (Primer-BLAST, NCBI, National Center for Biotechnology Information, https://www.ncbi.nlm.nih.gov/gene/?term=TLR, last access: 2 August 2021) were used to amplify the coding and flacking regions of the *Ovis aries* *KAP22-1* gene (GeneBank: *KX377618.1*). The PCR products (305 bp) were performed using thermal cycler PCR apparatuses (S1000 thermal cyclers, Bio-Rad, Hercules, CA, USA) in tubes containing 15 µL of PCR mixture containing the genomic DNA, 0.5 µL of each primer and 7.5 µL of Taq DNA polymerase (Intron bio, Germany) according to Williams et al. (1990). To amplify the target region, PCR conditions were used as follows: 2 min at 94 ${}^{\circ }$C, followed by 35 cycles of 30 s at 94 ${}^{\circ }$C, 30 s at 60 ${}^{\circ }$C and 30 s at 72 ${}^{\circ }$C, with a final extension of 5 min at 72 ${}^{\circ }$C. The amplified regions were detected and confirmed using the agarose gel electrophoresis. Then, the PCR products were prepared for genotyping and sequencing.

The single-strand conformation polymorphism (SSCP) technique was used to genotype the ovine *KAP22-1* in sheep. Briefly, a 15 µL aliquot of each amplicon was denatured at 95 ${}^{\circ }$C for 10 min; the samples were chilled on wet ice and immediately loaded onto 14 % acrylamide–bisacrylamide (37.5 : 1; Bio-Rad) gels. Electrophoresis for 16 h in 0.5X TBE buffer at 300 V was undertaken in Bio-Rad Protean II xi cells with water circulation at 18 ${}^{\circ }$C. The gels were silver-stained using the method of Byun et al. (2009).

### 2.3 DNA sequencing

To identify the polymorphic SNPs, PCR amplicons representing different SSCP banding patterns were delivered to the Macrogen sequencing company (https://www.macrogen.com/en/mainSeoul, last access: 2 August 2021, South Korea) for sequencing in both directions according to the BigDye terminator protocol. Sequences for the forward primers were aligned against the reference sequences from NCBIdb (*NC_056054*) for the corresponding amplified region. Likewise, the alignment was conducted against the sequence of the reverse primer of the corresponding region to ensure that the identified SNP is real. Identification of SNPs was performed using the 4Peaks software (https://nucleobytes.com/4peaks/, last access: 2 August 2021) for the pairwise alignment.

### 2.4 Statistical analysis

Levels of the traits were adjusted according to the trait (i.e., binary or continuous). Each trait was treated in a different way as they differ in nature. For the continuous traits (GFW, SL, FL, CR, FDsd and FD): a linear regression was fitted using a general linear model (GLM) to estimate the effect of the genotypes on the continuous trait. For the binary traits, distinct levels (2–5) were assigned, and the logistic regression process using the GENMOD procedure (*logit* and *cumlogit* link functions) in SAS (2004) was used to test the association of the binary and categorical subjective wool traits of KS, HG, GCG, BS, LS and SST. The following model was used:

$$Y_{ijkL}=\mu +G_{i}+H_{k}+B_{L}+e_{ijkL},$$

where $Y_{ijk}$ is the trait of interest, μ is the overall mean and G${}_{i}$ is the fixed effect of the ith genotype (AA, AB and BB). Genotypes with frequencies less than 5 % (i.e., AC, BC and CC) were excluded from the association analysis. $H_{k}$ is the fixed effect of the kth age of the animal (five levels: 1, 2, 3, 4, 5): the first level is animals less than 2 years old, the second level is animals from 2 to 4 years old, the third level is animals between 4 and 6 years old, the fourth level is animals from 6 to 8 years old and the fifth level is animals more than 8 years old. $B_{L}$ is the fixed effect of the breed (three levels), and $e_{ijkL}$ is random error. The random error was assumed to be normally distributed with a mean equal to zero, and variance is equal to $\delta_{e}^{2}$.

## 3 Results

### 3.1 *KAP22-1* variation in Egyptian sheep

**Figure 1:**
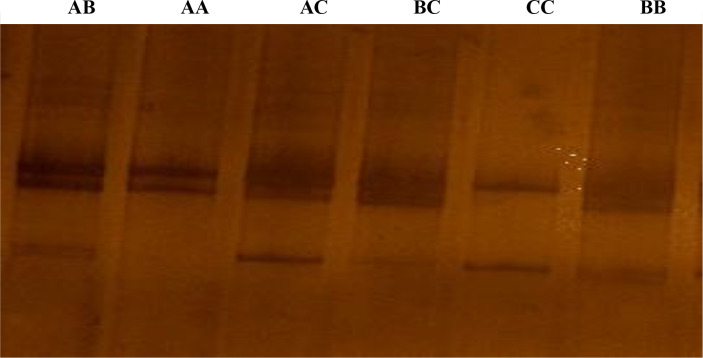
The SSCP conformation patterns of the ovine *KAP22-1* in Egyptian sheep. Three different alleles were identified (A, B and C).

**Figure 2:**
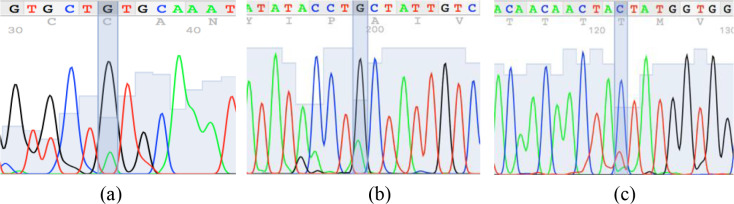
Sequence chromatogram of the amplified region of the ovine *KAP22-1* in Egyptian sheep showing the identified single-nucleotide polymorphisms (A, B and C).

According to the confirmation patterns of the PCR-SSCP, five patterns (five genotypes) and three alleles (named A, B and C) were observed for *KAP22-1* in the investigated individuals (Fig. 1). Generally, frequencies of the corresponding alleles in the whole studied population (i.e., regardless the breed) were about 48 %, 51 % and 1 % (Table 2). However, the C allele was observed exclusively in the Ossimi breed. The direct sequencing of PCR amplicons representing the three SSCP patterns identified three SNPs located at the 5${}^{\prime }$ upstream region of the ovine *KAP22-1* gene (Fig. 2). Two SNPs were novel (C > T: CHR1:123371431 and G > A: CHR1:123371508), and the other one was named *rs601417696* (G > A, 1:123371344).

**Table 2:** Genotypic and allelic frequencies of the amplified region of the ovine *KAP22-1* in Egyptian sheep breeds.

Breed	Allele frequency			Genotype frequency				
	A	B	C	AA	AB	BB	BC	CC
Barki	0.48	0.52	0	0.2	0.58	0.22	0	0
Rahmani	0.45	0.55	0	0.31	0.33	0.36	0	0
Ossimi	0.44	0.41	0.15	0.28	0.36	0.18	0.07	0.11
Total${}^{*}$	0.48	0.51	0.01	0.22	0.54	0.23	0.007	0.01

${}^{*}$ Frequency in the whole studied population.

### 3.2 Effect of the breed on wool traits

The breed of the animal had a significant effect (P<0.05) on SL, MF, PF, FD, SD${}_{\mathrm{fd}}$, CR, LG, KS, GCG and SST (Table 3). Ossimi sheep tended to significantly produce wool with longer SL (8.52 ± 0.59 cm), lower PF (20.18 ± 3.12 %), finer wool (FD = 26.24 ± 1.38 µm), lower SD${}_{\mathrm{fd}}$ (12.59 ± 2.17 µm), yellow (0.04 ± 0.49) and higher KS (1.6 ± 0.49) compared to those estimated in Barki and Rahmani sheep breeds. Comparably, wool produced from Rahmani sheep had the shortest SL (7.28 ± 0.47 cm), lowest MF (8.89 ± 2.41 %), highest PF (33.16 ± 2.79 %), highest FD (30.95 ± 1.24 µm) and lowest CR (0.63 ± 0.06). Likewise, Barki wool had the highest MF (14.31 ± 2.09 %), highest SD${}_{\mathrm{fd}}$ (16.55 ± 1.68 µm), highest CR (1.1 ± 0.05), highest KS and lowest LG and the whiter wool (1.55 ± 0.39).

**Table 3:** Effect of breed on the studied wool traits in Egyptian sheep${}^{a}$.

Traits${}^{b}$	Breed			P value
	Barki (206)	Rahmani (28)	Ossimi (23)	
GFW, g	1021.25 ± 44.53	NA	NA	NA
SL, cm	7.66 ± 0.41	7.28 ± 0.47	8.52 ± 0.59	0.013
MF%	14.31 ± 2.09	8.89 ± 2.41	11.89 ± 2.70	0.05
PF%	22.74 ± 2.42	33.16 ± 2.79	20.18 ± 3.12	0.001
FD, µm	26.92 ± 1.07	30.95 ± 1.24	26.24 ± 1.38	0.0001
SD${}_{\mathrm{fd}}$, µm	16.55 ± 1.68	16.27 ± 1.94	12.59 ± 2.17	0.042
CR%	1.10 ± 0.05	0.63 ± 0.06	0.77 ± 0.07	0.001
KS	-2.30 ± 0.4	-1.6 ± 0.49	1.6 ± 0.49	0.001
HG	-0.025 ± 0.31	0.74 ± 0.42	-0.77 ± 0.34	0.07
LG	-1.36 ± 0.35	-0.75 ± 0.45	-0.61 ± 0.35	0.003
BG	-0.17 ± 0.33	-0.02 ± 0.43	0.029 ± 0.43	0.8
GCG	1.51 ± 0.39	NA	0.04 ± 0.49	0.004
SST	-2.11 ± 0.44	-0.79 ± 0.58	-1.31 ± 0.4	0.001

${}^{a}$ Predicted least-square means and standard errors from GLMs. The significance P values < 0.05 are in bold. ${}^{b}$ NA: not available; GFW: greasy fleece weight (g); SL: staple length (cm); PF: prickle factor; MF: modulated fiber; FD: fiber diameter (µm); SD${}_{\mathrm{fd}}$: the standard deviation of FD; KS: kemp score; HG: handle grade; CR: crimp percentage %; GCG: greasy color grade; BG: bulk grade; LG: luster grade; SST: staple structure.

### 3.3 Effect of the animal age on wool traits

The age of the animal has a significant effect (P<0.05) on GFW, SL, FD${}_{\mathrm{sd}}$, CR, KS, LG and SST (Table 4). Animals less than 2 years old tended to produce more GFW (12 ± 0.44 kg) with higher SD${}_{\mathrm{fd}}$ (17.95 ± 1.97 µm) compared to older animals. The wool produced from animals between 2 and 4 years old was longer (SL = 8.39 ± 0.48 cm) and had higher CR (1.17 ± 0.43 %). Likewise, KS (1.99 ± 0.61), LG (0.72 ± 0.61) and SST (1.72 ± 0.65) were higher in animals more than 8 years old. Otherwise, animal age had no significant effect on the other wool traits.

**Table 4:** Predicted least-square means and standard errors of the effect of age of ewe on the studied wool traits in Barki sheep${}^{a}$.

Traits${}^{b}$	Age of ewe (N)					P value
	First (76)	Second (72)	Third (28)	Fourth (6)	Fifth (12)	
GFW, g	1208.96 ± 44.37	1135.02 ± 49.00	1150.06 ± 78.04	661.96 ± 154.77	953.14 ± 107.79	0.007
SL, cm	8.14 ± 0.48	8.39 ± 0.48	8.34 ± 0.43	7.01 ± 0.40	7.21 ± 0.66	0.002
MF%	12.14 ± 2.45	9.62 ± 2.47	14.04 ± 2.24	12.63 ± 2.06	10.06 ± 3.37	0.15
PF%	26.99 ± 2.84	26.88 ± 2.85	26.48 ± 2.59	21.82 ± 2.38	24.63 ± 3.91	0.27
FD, µm	28.75 ± 1.25	27.70 ± 1.28	28.66 ± 1.15	27.21 ± 1.05	27.87 ± 1.73	0.38
SD${}_{\mathrm{fd}}$, µm	17.95 ± 1.97	13.65 ± 1.99	14.85 ± 1.8	14.75 ± 1.66	14.58 ± 2.72	0.006
CR%	1.10 ± 0.40	1.17 ± 0.43	0.87 ± 0.29	0.76 ± 0.32	0.9 ± 0.23	0.006
KS	0.63 ± 0.58	1.09 ± 0.58	1.40 ± 0.59	1.95 ± 0.62	1.99 ± 0.61	0.001
HG	-0.16 ± 0.55	-0.72 ± 0.55	-0.23 ± 0.55	-0.18 ± 0.56	-0.26 ± 0.54	0.36
LG	-0.65 ± 0.63	0.11 ± 0.63	0.32 ± 0.63	0.68 ± 0.65	0.72 ± 0.61	0.006
BG	-1.08 ± 0.64	-0.96 ± 0.64	-1.14 ± 0.64	-1.00 ± 0.66	-0.86 ± 0.71	0.46
GCG	0.73 ± 0.54	0.61 ± 0.54	0.52 ± 0.56	-0.06 ± 0.58	-0.02 ± 0.53	0.25
SST	0.37 ± 0.65	0.24 ± 0.65	1.01 ± 0.66	1.67 ± 0.69	1.72 ± 0.65	0.013

${}^{a}$ The significance P values < 0.05 are in bold. ${}^{b}$ GFW: greasy fleece weight (g); SL: staple length (cm); MF: modulated fiber; PF: prickle factor; FD: fiber diameter (µm); SD${}_{\mathrm{fd}}$: the standard deviation of FD; CR: crimp percentage %; KS: kemp score; HG: handle grade; LG: luster grade; BG: bulk grade; GCG: greasy color grade; SST: staple structure.

### 3.4 Effect of *KAP22-1* genotypes on wool traits

Least-square means and standard errors for the effects of *KAP22-1* genotypes on the studied wool traits in Egyptian sheep are shown in Table 5. Genotypes and alleles with frequencies less than 5 % were excluded from the association tests. Conversely, animals with BB (n=44) genotypes produce wool with longer SL (8.38 ± 0.2 cm) followed by AA (8.27 ± 0.2 cm) and AB (7.95 ± 0.11 cm) genotypes. Additionally, CR and KS were significantly increased in animals with BB genotypes. A similar effect was observed on GCG as the AA animals produced lustrous wool (0.40 ± 1.7). Notably, animals with the AB genotype significantly (p=0.01) decreased the KS (0.39 ± 1.84) in the wool compared to other genotypes.

**Table 5:** Predicted least-square means and standard errors of the effect of *KAP22-1* genotypes on the studied wool traits in Egyptian sheep${}^{a}$.

Traits${}^{b}$	Genotypes			P value
	AA (40)	AB (111)	BB (43)	
GFW, g	1054.76 ± 47.7	1141.53 ± 27.11	1163.63 ± 65.91	0.28
SL, cm	8.27 ± 0.20	7.95 ± 0.11	8.38 ± 0.20	0.012${}^{*}$
MF%	12.6 ± 1.00	13.55 ± 0.56	12.49 ± 1.04	0.77
PF%	26.19 ± 1.2	26.35 ± 0.68	24.57 ± 1.24	0.78
FD, µm	26.43 ± 0.52	26.99 ± 0.29	26.80 ± 0.54	0.20
SD${}_{\mathrm{fd}}$, µm	13.69 ± 0.83	14.89 ± 0.47	14.85 ± 0.86	0.51
CR%	0.97 ± 0.03	7.95 ± 0.11	8.38 ± 0.21	0.01${}^{*}$
KS	0.45 ± 1.87	0.39 ± 1.84	1.98 ± 1.88	0.01${}^{*}$
HG	-0.018 ± 1.68	0.54 ± 1.66	0.58 ± 1.69	0.74
LG	-0.41 ± 1.87	0.22 ± 1.83	-0.12 ± 1.87	0.73
BG	0.71 ± 1.76	1.18 ± 1.73	0.98 ± 1.76	0.75
GCG	0.40 ± 1.73	0.25 ± 1.70	-0.62 ± 1.74	0.01${}^{*}$
SST	-22.62 ± 0.58	-23.49 ± 0.47	-22.86 ± 0.0	0.1

${}^{a}$ The significance P values < 0.05 are in bold. ${}^{b}$ GFW: greasy fleece weight (g); SL: staple length (cm); MF: medullated fiber; PF: prickle factor; FD: fiber diameter (µm); SD${}_{\mathrm{fd}}$: the standard deviation of FD; CR: crimp percentage %; KS: kemp score; HG: handle grade; LG: luster grade; BG: bulk grade; GCG: greasy color grade; SST: staple structure.

## 4 Discussion

Limited information is available about the genetic contribution of wool characteristics of the Barki sheep and the Nile valley breeds (e.g., Rahmani and Ossimi). This study reported significant differences in wool characteristics among the major sheep breeds in Egypt. Barki sheep produce lustrous, finer wool with higher medullation and crimp percentages and slightly lower KS and SST. In comparison, Rahmani have more colored wool with coarse FD, the lowest medullation and SST, and the highest PF and FD. Likewise, wool produced from Ossimi sheep was characterized by higher SL and KS and tended to be lustrous. However, FD was higher in Rahmani wool, but Barki wool may be the best as it had low PF and KS and was whiter, which means higher dying flexibility during manufacturing. Therefore, it is expected that Barki fleece is more economically valuable compared to Rahmani and Ossimi fleece. The abovementioned characteristics indicate that a wide range of wool types are produced from the local Egyptian sheep (El-Gabbas, 1999). The mixed structure (i.e., fine and coarse) fibers found in the same fleece was also reported in Egyptian wool. Therefore, it is recommended to assess the wool subjectively and objectively (Helal et al., 2019).

Generally, the amplified fragment of the *KAP22-1* gene tended to be polymorphic in the studied Egyptian sheep population as three alleles were identified (A, B and C). This result is consistent with other findings on *KAP* family genes (Li et al., 2017; Tao et al., 2017; Farag et al., 2018). Conversely, higher (Farag et al., 2018; Ullah et al., 2020a, 2021) and lower (Ullah et al., 2020b; Sallam et al., 2021) numbers of alleles were identified in other *KAP* genes and different sheep populations. *KRTAP6-5* was the most polymorphic gene in this family, which was identified in goats with 12 variants (Li et al., 2021). However, the C allele was less common, with frequency less than 5 % in the current Egyptian population, similar minor allele frequency was identified in *KAP21-1* in Merion × Southdown crosses (Li et al., 2017). This suggests that increasing the sample size in further investigations may succeed in identifying this allele.

Interestingly, allele A was identified as the common allele in the Ossimi breed that is recognized as producing whiter wool compared to other breeds (El-Gabbas, 1999). The presence of this allele significantly increased GCG in the wool (+0.40±1.73, P=0.01). Notably, allele B seemed to be important in wool production and characteristics. Animals that carry BB genotypes produce GFW more than non-carriers (1163.63±65.91 g versus 1054.76±47.7 g); however, this effect was statistically not significant. Furthermore, the wool produced from animals with BB genotypes had longer staples compared to other genotypes (8.38±0.20 cm versus 8.27±0.20 cm). This is consistent with the high frequency of the B allele in the studied population, which indicates that breeders may tend to select for it as a desirable allele to increase GFW (Asif et al., 2017). Likewise, it means that selection for the B allele at an early age may be worth it to increase wool production (Sallam et al., 2021). Conversely, wool produced from AA carriers had lower KS (0.45±1.87 versus 1.98±1.88, P=0.01) and increased the whiteness of the wool (+0.40±1.73 versus -0.62±1.74) compared to other genotypes.

So far, no information is available about the effect of *KAP22* polymorphisms on subjective wool production traits in sheep worldwide. Accordingly, it is hard to compare findings of this study with results of other reports. Nevertheless, variants in a different gene from the same family (i.e., *KAP6-1*) were associated with GCG and SST in Barki sheep (Sallam et al., 2021). Results of this study suggest that genetic markers may significantly influence subjective wool traits as well as objective measurements and should be considered. As expected, animals that carry the BB genotype increased the desirable wool yield (i.e., GFW), but it was accompanied by an increase in the unfavorable KS (+1.98±1.88). This confirms the negative correlation between both wool traits. Such information is important to be considered in breeding programs for wool production.

## 5 Conclusion

It is notable that Egyptian sheep produce a wide diverse wool type even in the same individual. This may be useful commercially in blending different wool types to make an appropriate composite for the carpet industry. In the current study, genetic polymorphisms were detected in the *KAP22-1* gene, and they strongly affect multiple wool characteristics. Significant associations were identified between the *KAP22-1* variants and CR (P=0.01), SL (P=0.012), SD${}_{\mathrm{fd}}$ (P=0.018), KS (P<0.001) and GCG (P=0.01). Interestingly, animals with BB genotypes tended to produce more wool yield (1163.63±65.91) with high SL (8.38±0.20), CR (8.38±0.21) and KS (1.98±1.88). Hence, the *KAP22-1* gene is a putative genetic marker for wool traits in the Egyptian sheep.

## Data Availability

The data are available from the corresponding author upon request.
